# Joint effects and dynamic trajectories of metabolic insulin resistance and systemic inflammation in the risk of renal cell carcinoma: a UK Biobank cohort analysis

**DOI:** 10.1007/s00345-026-06487-x

**Published:** 2026-05-28

**Authors:** Shuang Chen, Feipeng Jiang, Jie Wang, Jinlong Li, Wanlong Tan, Jianjun Ren, Meixia Zhang, Dechao Feng

**Affiliations:** 1 Department of Urology, Nanfang Hospital, Southern Medical University, Guangzhou, 510515 Guangdong PR China; 2 Department of Ophthalmology and Laboratory of Macular Disease, West China Hospital, Sichuan University, Chengdu, 610041 Sichuan China; 3 Urology & Nephrology Center, Department of Urology, Zhejiang Provincial People’s Hospital(Affiliated People’s Hospital), Hangzhou Medical College, Hangzhou, 310014 Zhejiang China; 4 Department of Institute of Biotherapy, Institute of Biotherapy, School of Biotechnology, Southern Medical University, Guangzhou, 510515 Guangdong PR China; 5 Department of Otolaryngology-Head & Neck Surgery, West China Hospital, Sichuan University, Chengdu, 610041 Sichuan China; 6 Division of Surgery & Interventional Science, University College London, London, W1W 7TS UK; 7 Department of Ophthalmology and Research Laboratory of Macular Disease, West China Hospital, Sichuan University, Chengdu, 610041 China

**Keywords:** Renal cell carcinoma, Metabolic score for insulin resistance, Systemic inflammation response index, Cohort-based study, UK Biobank

## Abstract

**Background:**

Renal cell carcinoma (RCC) is a significant urological malignancy with a rising incidence, increasingly linked to metabolic dysregulation and chronic systemic inflammation. While traditional metrics such as body mass index (BMI) are commonly used, they may not fully capture the biological heterogeneity underlying carcinogenesis. This study investigated the associations of the Metabolic Score for Insulin Resistance (METS-IR) and the Systemic Inflammation Response Index (SIRI) with subsequent RCC risk, together with their joint effects and longitudinal trajectory patterns.

**Methods:**

We conducted a retrospective analysis within the UK Biobank prospective cohort, comprising 410,766 participants aged 37–73 years. METS-IR and SIRI were calculated from baseline blood samples. Incident RCC was ascertained through national cancer registries. Multivariable Cox proportional hazards models were used to estimate hazard ratios (HRs) and 95% confidence intervals (CIs), because the outcome was time to incident RCC with variable follow-up and right censoring. Nonlinear relationships were evaluated using restricted cubic splines, and joint effects were assessed on an additive scale. Dynamic trajectory analysis based on repeat assessment data was treated as exploratory.

**Results:**

During a median follow-up of 13.65 years, 1,752 (0.43%) participants developed RCC, with a median time to diagnosis of 8.01 years among cases. Both biomarkers were independently associated with RCC risk. In fully adjusted models, each 1-SD increase in METS-IR was associated with a 26% higher RCC risk (HR: 1.26; 95% CI: 1.12–1.42), showing a linear dose-response pattern. SIRI showed a non-linear association, with risk increasing more sharply beyond an index value of approximately 1.2; participants in the highest quartile had a 57% higher risk (HR: 1.57; 95% CI: 1.35–1.83)than those in the lowest quartile. Participants with concomitantly high METS-IR and high SIRI had the highest risk (HR: 2.40; 95% CI: 2.06–2.79), although additive interaction metrics did not show statistical evidence of interaction. In exploratory trajectory analyses, persistently high METS-IR or SIRI was associated with higher RCC risk, whereas estimates for improved and worsened groups were more imprecise.

**Conclusion:**

METS-IR and SIRI were independently associated with RCC risk in this cohort. Their combined assessment may improve risk stratification. The findings further suggest that metabolic and inflammatory trajectory patterns may carry different prognostic information, although these longitudinal results should be interpreted cautiously and not as evidence of causality or risk reversibility.

**Graphic abstract:**

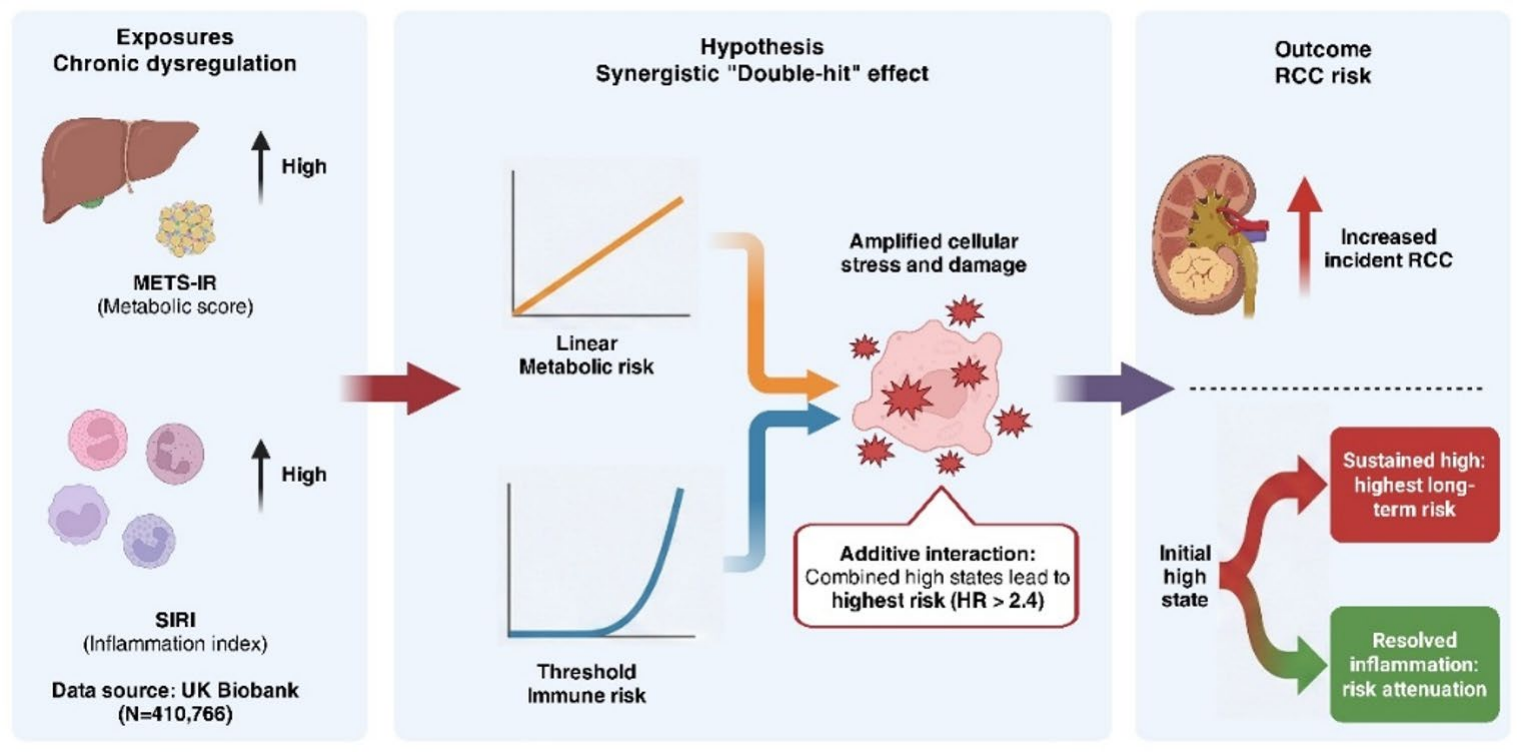

**Supplementary Information:**

The online version contains supplementary material available at 10.1007/s00345-026-06487-x.

## Introduction

 Renal cell carcinoma (RCC) represents a substantial burden on global public health, ranking as the sixth and ninth most common new cancer diagnosis for men and women, respectively, with approximately 434,000 new cases and 180,000 deaths reported annually worldwide [1–3]. This malignancy accounts for the majority of kidney cancer diagnoses and exhibits a steadily increasing incidence rate over recent decades, with global incident cases rising sharply from 160,000 in 1990 to 390,000 in 2021 [4]. The etiology of RCC is multifactorial, involving genetic, environmental, and lifestyle influences, yet emerging evidence strongly implicates modifiable lifestyle factors and their downstream physiological consequences—specifically, metabolic dysfunction and chronic systemic inflammation—as critical drivers of renal carcinogenesis [5–7]. For instance, epidemiological studies have linked obesity to a heightened RCC risk through mechanisms such as altered adipokine secretion, hormonal imbalances, and increased oxidative stress [8]. While obesity is a well-established risk factor, with meta-analyses indicating a 24% increased risk in men and 34% in women for every 5 kg/m² increment in BMI [8], reliance on Body Mass Index (BMI) alone is increasingly recognized as insufficient, as it fails to capture the visceral adiposity and insulin resistance that characterize the “metabolically obese normal-weight” phenotype, where up to one-third of individuals with normal BMI exhibit metabolic dysfunctions like dyslipidemia and hyperglycemia that elevate cancer susceptibility [9]. Consequently, there is a pressing need for more granular composite biomarkers that can quantify these underlying pathological states with greater precision, enabling better risk stratification and targeted prevention strategies.

Insulin resistance is a central mechanism linking metabolic syndrome to malignancy and may promote tumorigenesis through the insulin-like growth factor-1 (IGF-1) pathway, whereby hyperinsulinemia increases free IGF-1 levels and may stimulate cell proliferation while inhibiting apoptosis in renal cells [10]. The Metabolic Score for Insulin Resistance (METS-IR) is a practical non-insulin-based index integrating fasting glucose, triglycerides, HDL cholesterol, and BMI into a single metric, and correlates with established measures of insulin sensitivity [11, 12]. It has shown predictive value for metabolic and cardiovascular outcomes in prior longitudinal studies [13]. Yet, its association with RCC remains insufficiently characterized.

Parallel to metabolic dysregulation, chronic systemic inflammation creates a pro-tumorigenic microenvironment that facilitates cell proliferation, angiogenesis, and immune evasion through the release of cytokines and growth factors [14]. The Systemic Inflammation Response Index (SIRI), calculated from peripheral blood neutrophil, monocyte, and lymphocyte counts, offers a comprehensive view of the immune landscape by integrating pro-inflammatory (neutrophils and monocytes) and anti-inflammatory (lymphocytes) components, thereby reflecting the balance of host inflammatory responses more holistically than isolated markers like the neutrophil-to-lymphocyte ratio [15]. SIRI has shown superior predictive stability in various malignancies, including associations with poorer survival in pancreatic cancer patients post-chemotherapy, surpassing single-cell markers in prognostic accuracy [15].

Despite the biological plausibility linking these pathways to kidney cancer, few large-scale cohort studies have simultaneously evaluated METS-IR and SIRI in relation to RCC risk, and existing research has focused more broadly on metabolic syndrome components rather than these specific indices [16]. Furthermore, the potential interplay between metabolic and inflammatory processes remains insufficiently explored, as most investigations have not assessed their combined effects or interaction. Additionally, many epidemiological analyses rely on a single baseline measurement and therefore cannot address how longitudinal biomarker patterns relate to subsequent disease risk.

This study aimed to address these knowledge gaps using UK Biobank data. We sought to characterize the associations of METS-IR and SIRI with incident RCC, describe dose-response relationships, and quantify their combined effect. We also conducted an exploratory dynamic trajectory analysis to examine whether longitudinal changes in these biomarkers were associated with subsequent RCC risk.

## Methods

### Study population and data source

**Fig. 1:**
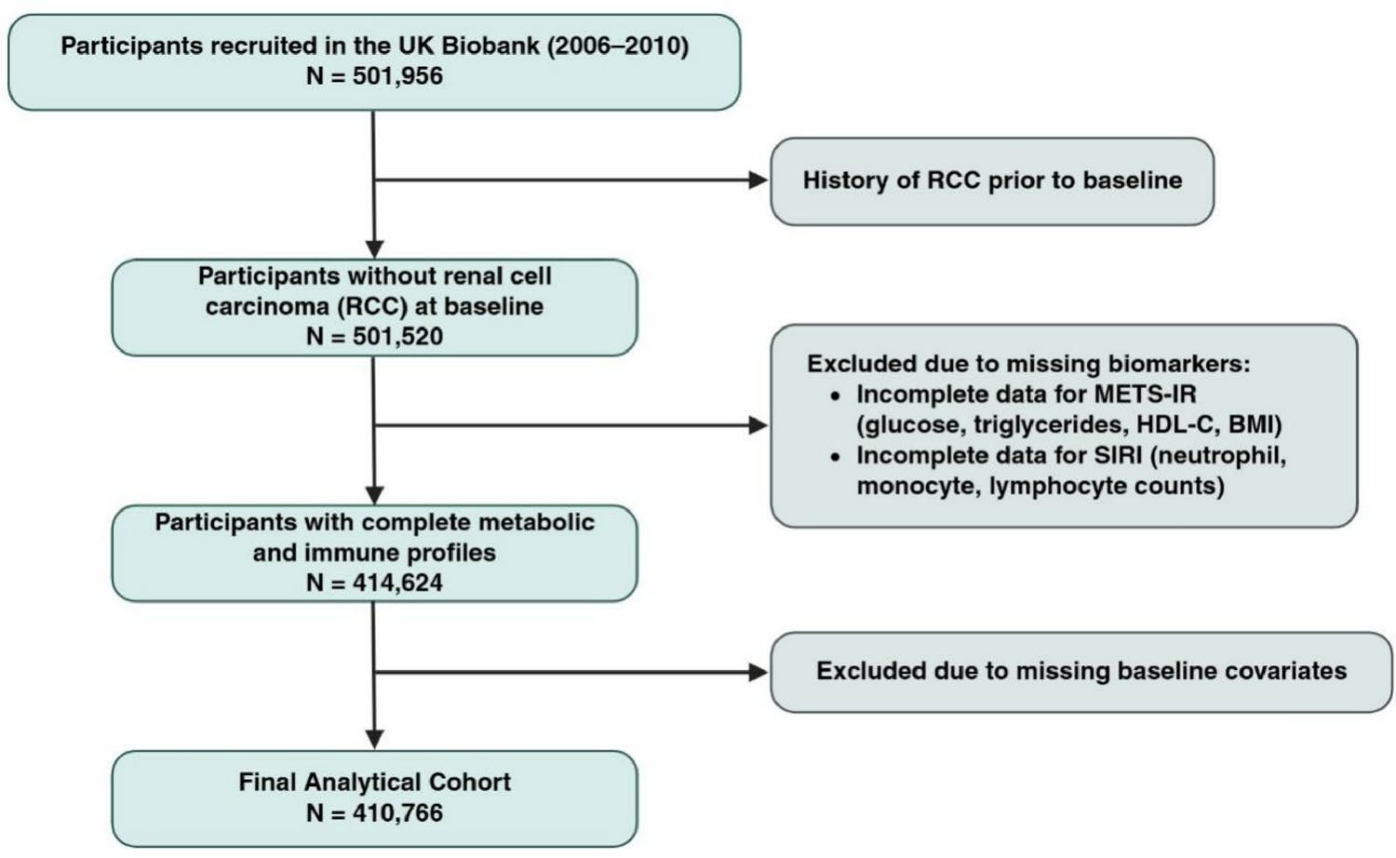
Flowchart of this study

This cohort analysis used data from the UK Biobank, a large-scale biomedical database that recruited over 500,000 participants aged 37–73 years across the United Kingdom between 2006 and 2010. All participants provided written informed consent, and the study protocol received ethical approval from the North West Multi-centre Research Ethics Committee. To establish the analytical cohort, we applied a strict stepwise exclusion process. Initially, participants with a history of RCC or any other malignancy prior to the baseline assessment, as well as those with zero or negative follow-up time, were excluded to minimize reverse causality (Fig. 1). Subsequently, we excluded individuals with missing data for essential metabolic biomarkers (fasting glucose, triglycerides, HDL cholesterol, and BMI) or immune markers (neutrophil, lymphocyte, monocyte, and platelet counts) necessary for calculating the exposure indices. We further excluded participants lacking sufficient data to calculate the baseline estimated glomerular filtration rate (eGFR), specifically serum creatinine, as well as those with missing information on key covariates, including ethnicity, Townsend deprivation index, smoking status, and fasting time. The final analytical cohort comprised 410,766 participants.

### Assessment of exposures

Blood samples were collected at baseline, and biochemical assays were performed using standard procedures documented in the UK Biobank showcase. The primary exposures of interest were the Metabolic Score for Insulin Resistance (METS-IR) and the Systemic Inflammation Response Index (SIRI). The METS-IR was calculated as ln[(2 × fasting glucose [mg/dL]) + triglycerides [mg/dL]] × BMI [kg/m²] / ln[HDL cholesterol (mg/dL)], serving as a composite marker for insulin resistance. The SIRI, reflecting systemic inflammation, was derived from peripheral blood cell counts using the formula: (neutrophil count × monocyte count) / lymphocyte count.

### Assessment of covariates

Baseline data regarding sociodemographic factors, lifestyle behaviors, anthropometric measurements, and medical history were collected through touchscreen questionnaires, nurse-led interviews, and physical measurements at assessment centers. Sociodemographic covariates included age at recruitment, sex, and ethnicity. Due to the limited sample size of minority groups, ethnicity was categorized into White and Others. Material deprivation was assessed using the Townsend Deprivation Index, a composite measure based on participant postcodes, with higher scores indicating greater deprivation. BMI was calculated as weight in kilograms divided by the square of height in meters based on measurements taken at the assessment center. Lifestyle factors, including smoking status and alcohol consumption, were categorized into never, previous, and current users. Clinical history of hypertension and diabetes was ascertained through self-reported diagnoses, nurse interviews, or the use of relevant medications (antihypertensives, insulin, or oral hypoglycemic agents). Information on the regular use of aspirin and non-steroidal anti-inflammatory drugs (NSAIDs) was also recorded via nurse-led interviews. To assess baseline kidney function, we calculated the eGFR using the Chronic Kidney Disease Epidemiology Collaboration (CKD-EPI) equation based on baseline serum creatinine levels.

### Outcome ascertainment

The primary outcome was incident RCC, defined according to the International Classification of Diseases, 10th Revision (ICD-10) code C64. Cancer diagnoses were ascertained through linkage to the national cancer registries of England, Wales, and Scotland. Follow-up duration was calculated from the date of baseline assessment to the date of the first RCC diagnosis, death, loss to follow-up, or the administrative censoring date (October 31, 2022), whichever occurred first.

### Statistical analysis

Baseline characteristics were stratified by incident RCC status (Case vs. Non-Case). Given the large sample size, normality of continuous variables was assessed using the Shapiro-Wilk test on a random subset of 5,000 participants. Due to the skewed distribution of metabolic and immune biomarkers, continuous variables were expressed as medians with interquartile ranges (IQR) and compared using the Wilcoxon rank-sum test. Categorical variables were presented as frequencies and percentages, with differences assessed using Pearson’s Chi-squared test. Raincloud plots were generated using the ggdist and gghalves packages to visualize the distributions of metabolic and inflammatory biomarkers. To accommodate the skewness of immune markers, the Systemic Inflammation Response Index (SIRI) was visualized on a logarithmic$\:\:{\mathrm{l}\mathrm{o}\mathrm{g}}_{10}$ scale. Kaplan–Meier curves were generated according to quartiles of METS-IR and SIRI, and differences between groups were compared using the log-rank test. Because the primary outcome was time to incident RCC and follow-up duration varied across participants, time-to-event analysis using Cox proportional hazards models was preferred over logistic regression to account for censoring and event timing. All statistical analyses were performed using R software (version 4.1.3), and a two-sided P-value < 0.05 was considered statistically significant.

### Primary associations analysis

To investigate the prospective associations of metabolic and inflammatory indices with the risk of incident RCC, we employed multivariable Cox proportional hazards regression models to calculate hazard ratios (HRs) and 95% confidence intervals (CIs). The primary exposures, METS-IR and SIRI, were analyzed in two formats: first, as continuous variables standardized using Z-scores (mean = 0, standard deviation = 1) to estimate the risk associated with per 1-SD increase; and second, as categorical variables based on quartiles (Q1–Q4), with the lowest quartile serving as the reference group. To control for potential confounders, we constructed three sequential models. Model 1 adjusted for age and sex. Model 2 further adjusted for sociodemographic factors, including the Townsend deprivation index and ethnicity. The fully adjusted Model 3 additionally included BMI, smoking status, alcohol consumption, history of hypertension, history of diabetes, use of aspirin and NSAIDs, and baseline estimated glomerular filtration rate (eGFR). Crucially, given that BMI is an intrinsic component of the METS-IR algorithm, it was excluded from the covariates in Model 3 specifically for the analysis of METS-IR to prevent multicollinearity and over-adjustment. Tests for linear trend were performed by assigning the median value of each quartile to participants in that category and modeling this as a continuous variable.

### Dose-response analysis

To visualize the flexible dose-response relationships between the novel biomarkers and RCC risk, we employed restricted cubic splines (RCS) with four knots placed at the 5th, 35th, 65th, and 95th percentiles of the exposure distribution. The median value of each index served as the reference point (Hazard Ratio = 1). Consistent with the main Cox regression analysis, these models were adjusted for the full set of covariates in Model 3; specifically, BMI was included for the SIRI analysis but excluded for METS-IR to prevent over-adjustment. We formally tested for non-linearity using a likelihood ratio test by comparing the model with only the linear term to the model containing both linear and cubic spline terms, considering a P-value < 0.05 as indicative of a significant deviation from linearity.

### Joint effect and interaction analysis

To evaluate the combined impact of metabolic and inflammatory dysregulation on RCC risk, we performed a joint effect analysis by categorizing participants into four groups based on the median values of METS-IR and SIRI. These groups were defined as: Low-Risk (reference group, both markers < median), Metabolic-Risk only (METS-IR ≥ median, SIRI < median), Inflammatory-Risk only (METS-IR < median, SIRI ≥ median), and the Combined High-Risk group (both markers ≥ median).We further assessed biological interaction on the additive scale, which is widely considered more relevant for public health interventions than multiplicative interaction. Three standard indices were calculated: the Relative Excess Risk due to Interaction (RERI), the Attributable Proportion (AP), and the Synergy Index (SI). A RERI > 0, AP > 0, or SI > 1 would indicate a synergistic effect. The 95% confidence intervals (CIs) for these indices were estimated using the delta method to account for the covariance of the coefficients.

### Subgroup analysis

To verify the consistency of the associations, stratified analyses were conducted based on potential effect modifiers, including age (< 60 vs. ≥60 years), sex, BMI categories (normal weight, overweight, obese), smoking status (never vs. ever), diabetes, and hypertension. In these stratified models, METS-IR and SIRI were entered as standardized continuous variables (Z-scores) to facilitate comparison across strata. Potential effect modification was statistically evaluated by including a cross-product term (biomarker × subgroup) in the Cox proportional hazards models, utilizing likelihood ratio tests to assess significance.

Exploratory histology-specific subgroup analyses were conducted using linked cancer registry morphology data. For each subtype, cause-specific Cox proportional hazards models were fitted in the full analytic cohort, with the subtype of interest treated as the event and other incident kidney cancer subtypes censored at the time of diagnosis.

### Dynamic trajectory analysis

To investigate the impact of longitudinal changes in metabolic and inflammatory status on RCC risk and to assess the potential benefit of risk factor modification, we conducted a dynamic trajectory analysis using a landmark approach. The “landmark time” was defined as the date of the second assessment (repeat visit). Here, the repeat visit refers to the UK Biobank repeat assessment visit, a second longitudinal study visit unrelated to surgery or postoperative follow-up. The study population was strictly restricted to participants who attended the repeat visit and remained free of kidney cancer until this landmark time point. Participants were categorized into four phenotypic groups based on their biomarker status (High vs. Low, dichotomized by the baseline median) across the two time points: “Stable Low” (low at both baseline and repeat, serving as the reference); “Improved” (high at baseline, low at repeat); “Worsened” (low at baseline, high at repeat); and “Persistent High” (high at both baseline and repeat). Cox proportional hazards models were fitted using the same covariate structure as the fully adjusted primary analysis; specifically, models were adjusted for age, sex, Townsend deprivation index, ethnicity, smoking status, alcohol consumption, history of hypertension, history of diabetes, use of aspirin and NSAIDs, and baseline eGFR, with BMI additionally included for SIRI but excluded for METS-IR because it is a component of the METS-IR formula. Because the repeat-assessment subset was smaller and several estimates were imprecise, these analyses were interpreted cautiously.

### Sensitivity analysis

To verify the robustness of the primary findings and minimize the potential for reverse causation and confounding by pre-existing disease, we performed a series of rigorous sensitivity analyses. First, to eliminate the influence of prior malignancies and potential treatment-related metabolic or inflammatory alterations, we excluded participants who had been diagnosed with any malignancy (ICD-10 codes C00–C97) prior to the baseline assessment, with the exception of non-melanoma skin cancer. Second, to address the possibility of reverse causality where undiagnosed preclinical RCC might influence baseline biomarker levels, we conducted a lag-time analysis by excluding participants diagnosed with RCC within the first 24 months of follow-up. The Cox proportional hazards models were re-run on these restricted datasets using the fully adjusted Model 3 specifications, maintaining the distinct adjustment strategies for METS-IR (excluding BMI) and SIRI (including BMI). Third, to further assess the potential influence of lifestyle-related residual confounding, we additionally adjusted the fully adjusted Cox models for dietary and physical activity variables, including cooked vegetable intake, raw vegetable intake, fresh fruit intake, processed meat intake, water intake, and physical activity level categorized according to the International Physical Activity Questionnaire (IPAQ).

## Results

### Baseline characteristics

Among 410,766 participants, 1,752 (0.43%) developed incident RCC over a median follow-up of 13.65 years (Table 1). Among cases, the median time from baseline to RCC diagnosis was 8.01 years (IQR, 4.72–10.91). RCC cases were significantly older (median age, 62.0 vs. 58.0 years), more often male (65% vs. 46%), and more often White (97% vs. 95%) than non-cases.They also had higher smoking prevalence, lower baseline eGFR, and greater prevalence of hypertension (82.6% vs. 60.9%) and diabetes (18.8%vs. 10.3%). Cases exhibited a distinct metabolic and pro-inflammatory phenotype, with significantly elevated median BMI, METS-IR (42.54 vs. 38.91), and SIRI (1.15 vs. 0.96) (all *P* < 0.001) (Table 2).

**Table 1:** Baseline characteristics of the study population stratified by incident renal cell carcinoma status

Characteristic	Total (*N* = 410,766)	Non-case (*N* = 409,014)	Case (*N* = 1,752)	*P* value
Age (years)	58.0 (50.0, 63.0)	58.0 (50.0, 63.0)	62.0 (57.0, 66.0)	< 0.001
*Sex*				< 0.001
Female	221,034 (54%)	220,421 (54%)	613 (35%)	
Male	189,732 (46%)	188,593 (46%)	1,139 (65%)	
*Ethnicity*				< 0.001
White	389,581 (95%)	387,878 (95%)	1,703 (97%)	
Others	21,185 (5%)	21,136 (5%)	49 (3%)	
Townsend Deprivation Index	-2.17 (-3.66, 0.49)	-2.17 (-3.66, 0.49)	-2.31 (-3.75, 0.35)	0.136
BMI (kg/m²)	26.74 (24.14, 29.89)	26.74 (24.14, 29.88)	28.13 (25.51, 31.51)	< 0.001
*Smoking status*				< 0.001
Never	224,531 (55%)	223,747 (55%)	784 (45%)	
Previous	142,867 (35%)	142,153 (35%)	714 (41%)	
Current	43,368 (11%)	43,114 (11%)	254 (14%)	
*Alcohol consumption*				0.248
Never	17,797 (4%)	17,733 (4%)	64 (4%)	
Previous	14,636 (4%)	14,566 (4%)	70 (4%)	
Current	378,333 (92%)	376,715 (92%)	1,618 (92%)	
History of hypertension	250,462 (61%)	249,014 (61%)	1,448 (83%)	< 0.001
History of diabetes	42,279 (10%)	41,950 (10%)	329 (19%)	< 0.001
eGFR (mL/min/1.73 m²)	96.35 (86.04, 105.88)	96.37 (86.08, 105.90)	89.42 (78.34, 100.17)	< 0.001
Follow-up duration (years)	13.7 (12.9, 14.4)	13.7 (12.9, 14.4)	8.0 (4.7, 10.9)	< 0.001
Aspirin use	60,489 (15%)	60,069 (15%)	420 (24%)	< 0.001
NSAID use	71,272 (17%)	71,017 (17%)	255 (15%)	0.002
METS-IR index	38.92 (33.47, 45.46)	38.91 (33.46, 45.45)	42.54 (36.89, 49.33)	< 0.001
SIRI index	0.96 (0.68, 1.36)	0.96 (0.68, 1.36)	1.15 (0.81, 1.68)	< 0.001

Data are presented as median (Q1, Q3) for continuous variables and n (%) for categorical variables. Age and follow-up duration are reported to 1 decimal place; BMI, Townsend Deprivation Index, eGFR, METS-IR, and SIRI are reported to 2 decimal places; percentages are reported as whole numbers. P values were calculated using the Wilcoxon rank-sum test for continuous variables and Pearson’s chi-squared test for categorical variables

*BMI* body mass index; *eGFR* estimated glomerular filtration rate; *METS-IR* metabolic score for insulin resistance; *SIRI* systemic inflammation response index; *NSAID* non-steroidal anti-inflammatory drug; *RCC* renal cell carcinoma

**Table 2:** Association of metabolic and inflammatory indices with the risk of incident renal cell carcinoma

Exposure	Model 1		Model 2		Model 3	
	HR (95% CI)	*P* value	HR (95% CI)	*P* value	HR (95% CI)	*P* value
*METS-IR*						
Per 1-SD increase	1.35 (1.29–1.41)	< 0.001	1.35 (1.29–1.41)	< 0.001	1.26 (1.12–1.42)	< 0.001
*Quartiles*						
Q1 (Reference)	1.00 (Ref)		1.00 (Ref)		1.00 (Ref)	
Q2	1.45 (1.23–1.72)	< 0.001	1.46 (1.23–1.72)	< 0.001	1.25 (1.04–1.49)	0.014
Q3	1.68 (1.43–1.98)	< 0.001	1.68 (1.43–1.98)	< 0.001	1.25 (1.04–1.51)	0.020
Q4	2.36 (2.02–2.77)	< 0.001	2.37 (2.02–2.77)	< 0.001	1.40 (1.11–1.78)	0.005
P for trend		< 0.001		< 0.001		0.016
*SIRI*						
Per 1-SD increase	1.02 (1.01–1.03)	0.007	1.02 (1.01–1.03)	0.006	1.02 (1.01–1.03)	0.034
*Quartiles*						
Q1 (Reference)	1.00 (Ref)		1.00 (Ref)		1.00 (Ref)	
Q2	1.26 (1.07–1.48)	0.005	1.25 (1.06–1.47)	0.006	1.18 (1.01–1.39)	0.041
Q3	1.51 (1.30–1.76)	< 0.001	1.49 (1.28–1.74)	< 0.001	1.35 (1.15–1.57)	< 0.001
Q4	1.91 (1.65–2.21)	< 0.001	1.88 (1.62–2.18)	< 0.001	1.57 (1.35–1.83)	< 0.001
P for trend		< 0.001		< 0.001		< 0.001

Model adjustments: Model 1: Adjusted for age and sex. Model 2: Adjusted for age, sex, Townsend deprivation index, and ethnicity. Model 3: Adjusted for age, sex, Townsend deprivation index, ethnicity, smoking status, alcohol consumption, history of hypertension, history of diabetes, use of aspirin and NSAIDs, and baseline eGFR. Note: BMI was adjusted in Model 3 for the analysis of SIRI but was excluded from the analysis of METS-IR to avoid multicollinearity, as BMI is a component of the METS-IR calculation

*HR* hazard ratio; *CI* confidence interval; *METS-IR* metabolic score for insulin resistance; *SIRI* systemic inflammation response index; *BMI* body mass index; *eGFR* estimated glomerular filtration rate

### Association of metabolic and inflammatory indices with incident RCC

Participants who developed RCC had higher baseline METS-IR and SIRI values than those who did not (Figure S1). Specifically, in the fully adjusted analysis (Model 3), each 1-SD increase in METS-IR was associated with a 26% higher RCC risk (HR: 1.26; 95% CI: 1.12–1.42; *P* < 0.001). A positive dose-response pattern was confirmed across quartiles (P-trend = 0.016); participants in the highest quartile (Q4) had a 40% higher risk than those in Q1 (HR: 1.40; 95% CI: 1.11–1.78). For SIRI, each 1-SD increase showed a modest but statistically significant association in Model 3 (HR: 1.02; 95% CI: 1.01–1.03; *P* = 0.034). Quartile analysis revealed a stronger gradient (P-trend < 0.001), with Q4 associated with a 57% higher risk versus Q1 (HR: 1.57; 95% CI: 1.35–1.83). Supplementary Kaplan–Meier analyses showed clear separation of cumulative incidence curves across quartiles of both METS-IR and SIRI, with a graded increase from Q1 to Q4 (both log-rank *P* < 0.0001; Figure S2).

### Dose-response relationships

**Fig. 2:**
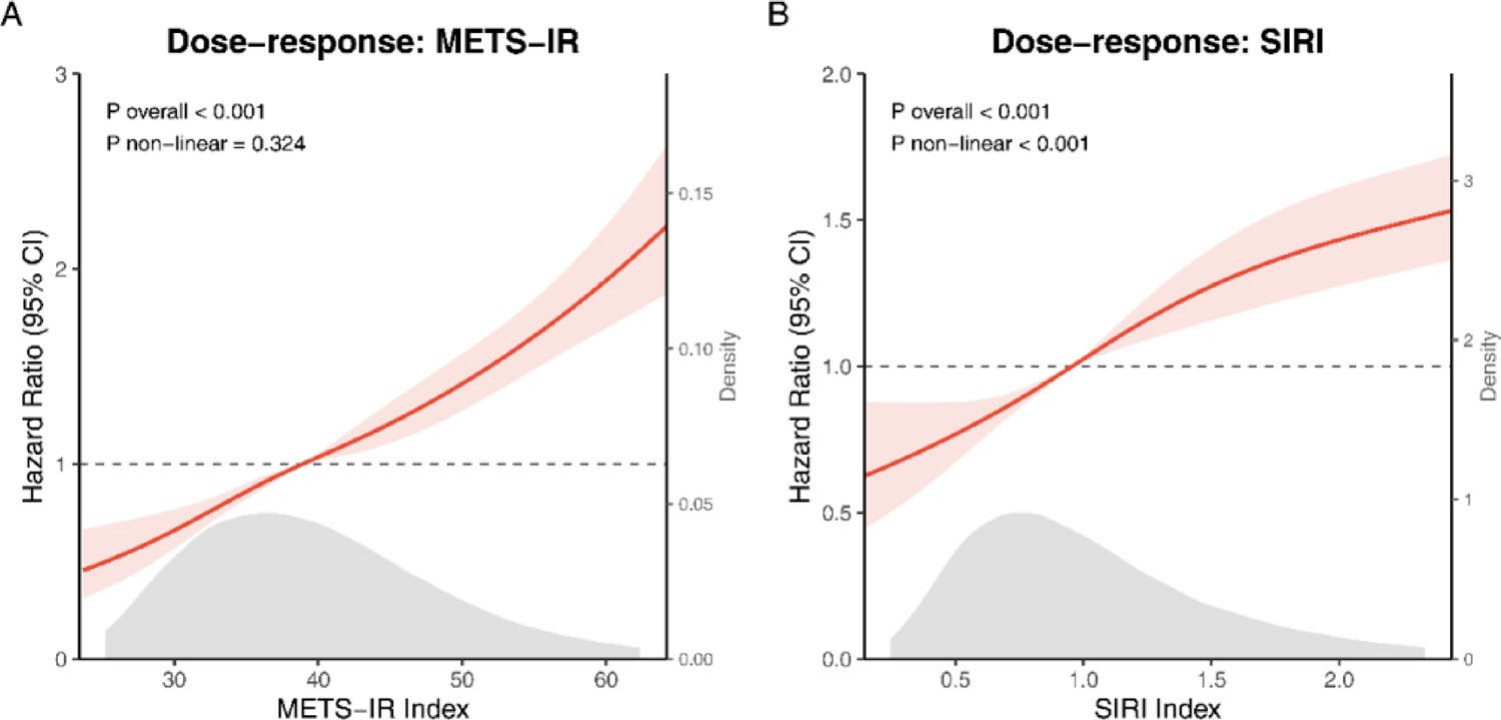
Dose-response associations of metabolic and inflammatory biomarkers with the risk of incident renal cell carcinoma. Restricted cubic spline (RCS) visualizations illustrate the estimated hazard ratios (solid lines) and corresponding 95% confidence intervals (shaded areas) for (**A**) the Metabolic Score for Insulin Resistance (METS-IR) and **B** the Systemic Inflammation Response Index (SIRI). Analyses were based on the fully adjusted Model 3, controlling for age, sex, Townsend deprivation index, ethnicity, smoking status, alcohol consumption, comorbidities (hypertension, diabetes), medication use (aspirin, NSAIDs), and baseline eGFR. Note that Body Mass Index (BMI) was included as a covariate for the SIRI analysis but was specifically excluded from the METS-IR model to avoid multicollinearity. The median value of each biomarker served as the reference point (HR = 1.0), indicated by the dashed horizontal line. The gray density plots along the x-axis depict the frequency distribution of the study population

Restricted cubic spline analysis showed a linear dose-response relationship for METS-IR (P-overall < 0.001, P-non-linear = 0.324), with RCC risk rising continuously. For SIRI, a significant non-linear, J-shaped relationship was observed (P-overall < 0.001, P-non-linear < 0.001) (Fig. 2). Risk remained stable below SIRI ~ 1.2, then escalated sharply at higher levels.

### Joint effect and additive interaction

**Fig. 3:**
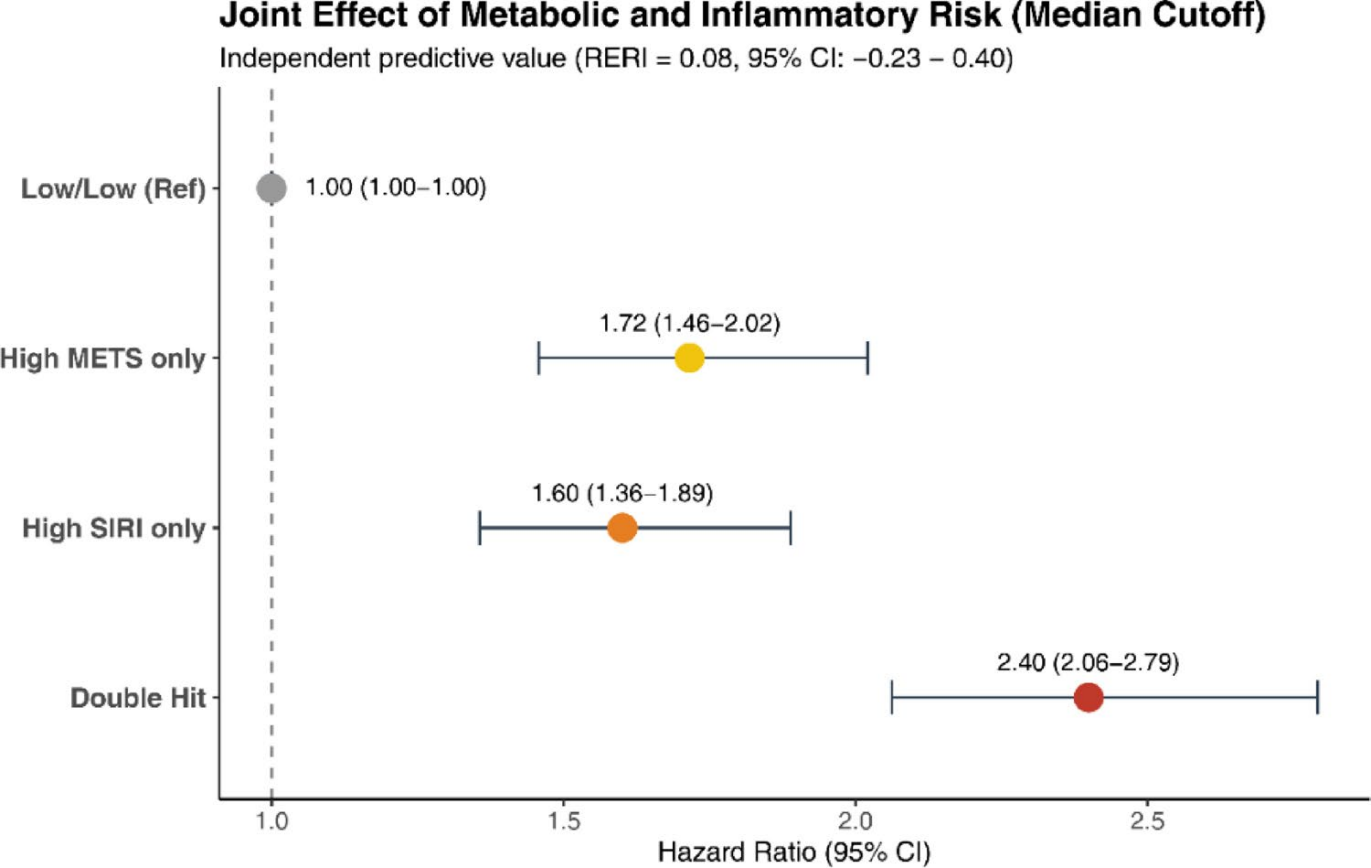
Joint associations of metabolic and inflammatory profiles with the risk of incident renal cell carcinoma. This forest plot illustrates the hazard ratios (HRs) and 95% confidence intervals for four phenotypic groups defined by the median thresholds of METS-IR and SIRI. The “Low/Low” group (both markers below the median) served as the reference category. The “Double-Hit” group (highlighted in red), characterized by simultaneous elevations in both metabolic and inflammatory markers, exhibited the highest cumulative risk (HR: 2.40). Indices of biological interaction on the additive scale, including the Relative Excess Risk due to Interaction (RERI), Attributable Proportion (AP), and Synergy Index (SI), are detailed in the embedded table

Compared to the Low-Risk group, isolated Metabolic-Risk and Inflammatory-Risk groups had elevated HRs of 1.72 (95% CI: 1.46–2.02) and 1.60 (95% CI: 1.36–1.89), respectively (Fig. 3).The Combined High-Risk group had the highest risk (HR: 2.40; 95% CI: 2.06–2.79) (Table S1). Measures of additive interaction (RERI: 0.08; 95% CI: -0.23 to 0.40) indicated cumulative, but not synergistic, effects (Table S1).

### Subgroup analysis

**Fig. 4:**
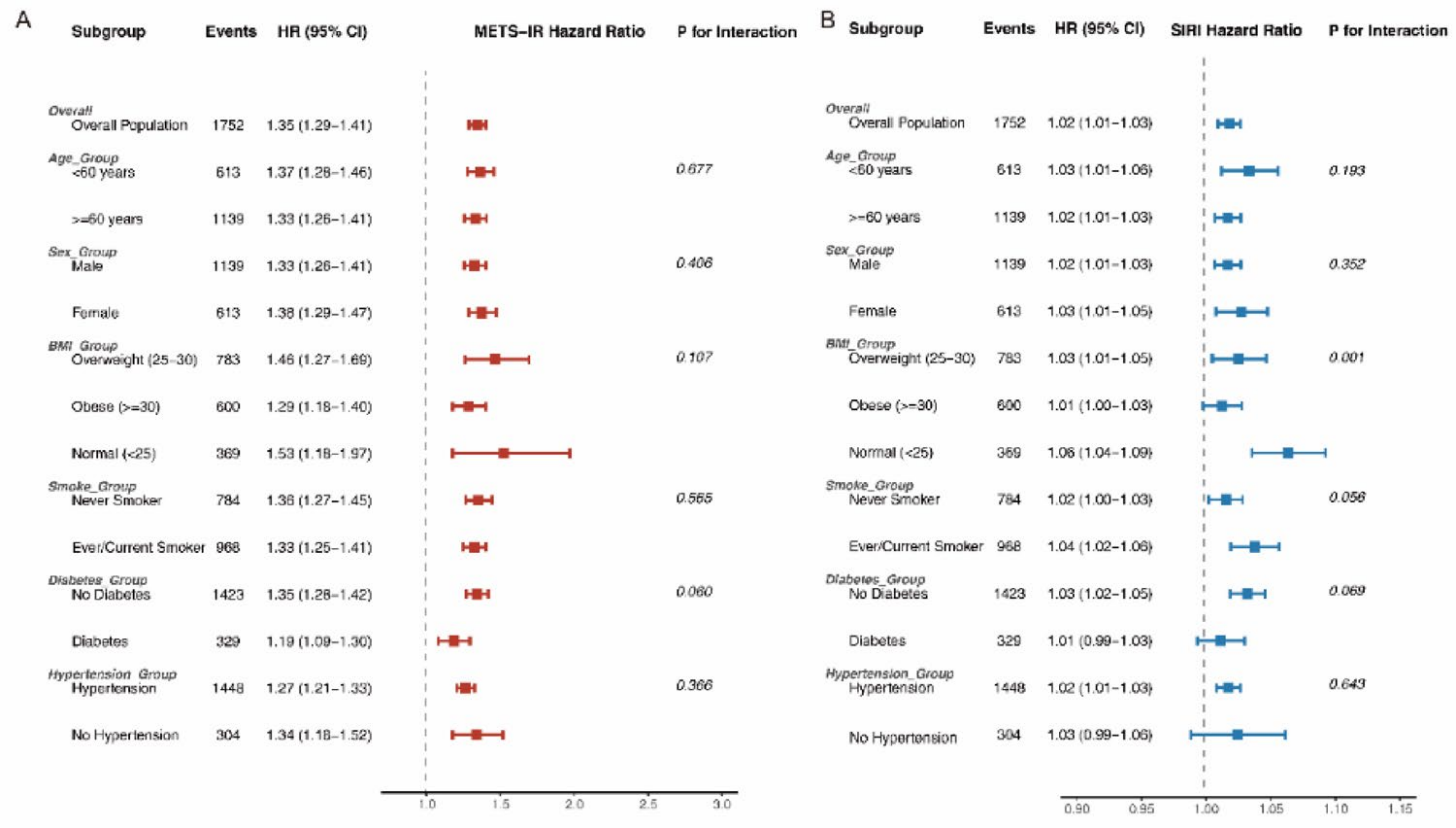
Stratified analyses of the associations between metabolic and inflammatory biomarkers and renal cell carcinoma risk. Forest plots display the hazard ratios (HRs) per 1-SD increase in exposure across pre-specified clinical subgroups. **A** The Metabolic Score for Insulin Resistance (METS-IR) demonstrates consistent risk elevation across all strata, with a notably stronger association observed in normal-weight participants compared to obese individuals (P for interaction < 0.05). **B** The Systemic Inflammation Response Index (SIRI) exhibits stable and independent predictive value across all demographic and clinical subgroups. P-values for interaction are provided for each stratification variable to assess potential effect modification

The positive association for METS-IR was consistent across all subgroups, with a stronger effect observed in participants with normal BMI (P-interaction < 0.05) (Fig. 4A). SIRI associations remained stable across subgroups, with no significant heterogeneity by age or sex (Fig. 4B).

Among 1,752 incident kidney cancer cases in the primary analysis, morphology information was available for 1,587 cases (90.6%). Because the morphology distribution was highly imbalanced and several categories were rare or non-specific (Figure S3), exploratory subtype-specific Cox analyses were restricted to clear-cell RCC and papillary RCC. In fully adjusted cause-specific Cox models, higher METS-IR was associated with greater risk of clear-cell RCC (HR 1.44, 95% CI 1.35–1.54; *P* < 0.001), whereas SIRI was not significantly associated with clear-cell RCC (HR 1.02, 95% CI 1.00–1.04; *P* = 0.300). Neither METS-IR nor SIRI was significantly associated with papillary RCC (METS-IR: HR 1.04, 95% CI 0.84–1.30; *P* = 0.700; SIRI: HR 1.02, 95% CI 1.00–1.05; *P* = 0.300) (Table S2).

### Longitudinal trajectories and risk reversibility

**Fig. 5:**
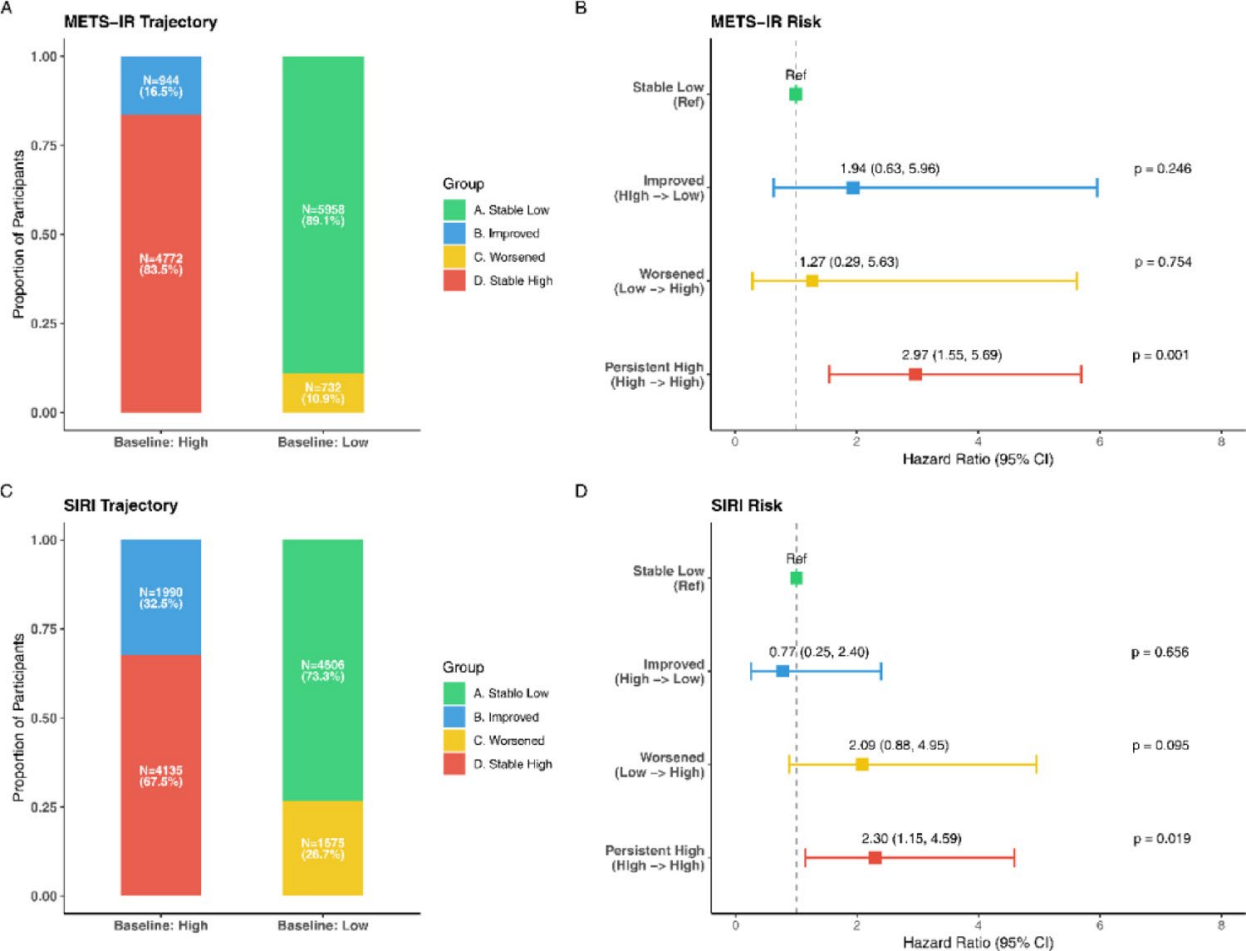
Impact of longitudinal changes in metabolic and inflammatory status on the risk of incident renal cell carcinoma (Landmark Analysis). **A**, **B** For METS-IR, the Persistent High group showed the clearest elevation in risk, whereas the Improved and Worsened groups had imprecise estimates with confidence intervals crossing the null. **C**, **D** For SIRI, the Persistent High group was associated with higher risk, while the Improved and Worsened groups should be interpreted cautiously because several estimates were imprecise. *HR* hazard ratio; *CI* confidence interval; *METS-IR* metabolic score for insulin resistance; *SIRI* systemic inflammation response index

For METS-IR, compared with the Stable Low group, the Improved group had an HR of 1.94 (95% CI: 0.63–5.96; *P* = 0.246), the Worsened group had an HR of 1.27 (95% CI: 0.29–5.63; *P* = 0.754), and the Persistent High group had the highest risk (HR: 2.97; 95% CI: 1.55–5.69; *P* = 0.001) (Fig. 5A-B). For SIRI, the corresponding HRs were 0.77 (95% CI: 0.25–2.40; *P* = 0.656), 2.09 (95% CI: 0.88–4.95; *P* = 0.095), and 2.30 (95% CI: 1.15–4.59; *P* = 0.019), respectively (Fig. 5C-D).

### Sensitivity analysis

After excluding participants with prior malignancy or early-diagnosis RCC, associations remained robust. For METS-IR, the per 1-SD HR was 1.29 (95% CI: 1.14–1.46; *P* < 0.001). For SIRI, participants in Q4 had a 53% higher risk versus Q1 (HR: 1.53; 95% CI: 1.31–1.79; *P* < 0.001). The above results were presented in Table S3. In an additional sensitivity analysis with further adjustment for dietary factors and physical activity, the associations also remained materially unchanged (Table S4). For METS-IR, each 1-SD increase was associated with a 43% higher RCC risk (HR: 1.43; 95% CI: 1.25–1.62; *P* < 0.001), and participants in Q4 had a 63% higher risk compared with those in Q1 (HR: 1.63; 95% CI: 1.25–2.13; *P* < 0.001; P for trend = 0.024). For SIRI, each 1-SD increase was associated with a 3% higher RCC risk (HR: 1.03; 95% CI: 1.02–1.05; *P* = 0.006), and participants in Q4 had a 64% higher risk compared with those in Q1 (HR: 1.64; 95% CI: 1.38–1.95; *P* < 0.001; P for trend < 0.001).

## Discussion

In this large-scale UK Biobank cohort analysis, higher METS-IR and SIRI were independently associated with greater RCC risk.The joint and trajectory analyses further suggest that concurrent metabolic and inflammatory dysregulation and persistently high biomarker patterns may identify less favorable risk profiles. These findings should be interpreted within an observational framework and not as evidence of causality, reversibility, or intervention benefit.

The linear dose-response relationship observed between METS-IR and RCC highlights the continuous nature of metabolic risk. Every 1-SD increase in METS-IR was associated with a 26% increase in RCC risk, a finding that underscores the importance of the insulin-IGF axis in tumor promotion [17]. Specifically, elevated METS-IR correlates with hyperinsulinemia, which enhances the bioavailability of IGF-1 by downregulating IGF-binding proteins, thereby promoting mitogenic signaling through IGF-1R activation in renal tubular cells, leading to increased proliferation and reduced apoptosis [5]. Notably, our subgroup analysis revealed a stronger association between METS-IR and RCC in participants with normal BMI compared to those with obesity. This paradoxical finding suggests that in the absence of overt obesity, insulin resistance acts as a silent but potent driver of malignancy, likely through hyperinsulinemia and the bioavailability of IGF-1, which inhibit apoptosis and stimulate cell proliferation [10]. For instance, studies have shown that insulin resistance can induce DNA damage and oxidative stress in renal cells, independent of adiposity, exacerbating oncogenic pathways [18]. Furthermore, such elevated oxidative stress is closely intertwined with dysregulated iron metabolism and evasion of ferroptosis—a key vulnerability in cancer progression—where transporters like SLC40A1 play a pivotal role [19]. This underscores the clinical necessity of screening for metabolic health beyond simple anthropometric measures like BMI, as METS-IR provides a more integrated assessment of glucose, lipid, and anthropometric derangements that traditional indices like HOMA-IR may overlook [13].

In contrast to the more linear metabolic risk pattern, the association between SIRI and RCC appeared non-linear, with a more pronounced increase in risk at higher values and a threshold-like inflection around an index value of approximately 1.2. Participants in the highest quartile had a 57% higher risk than those in the lowest quartile [20]. Although this pattern is compatible with the possibility that systemic inflammation becomes more relevant once a certain burden is reached, it should not be interpreted as proof of a biological “tipping point.” The composition of SIRI—elevated neutrophils and monocytes relative to lymphocytes—remains biologically plausible in the context of tumor-related inflammation and immune escape [21, 22]. For example, neutrophils may release reactive oxygen species and matrix metalloproteinases that facilitate extracellular matrix remodeling and vascular invasion, while monocyte-derived macrophages may polarize toward an M2 phenotype that suppresses T-cell responses, thereby contributing to a microenvironment permissive to tumor progression in RCC [23]. This interpretation is also broadly consistent with observations in other malignancies, where low-grade or moderate inflammation may differ biologically from chronic and sustained inflammatory activation [24]. However, the present observational data cannot establish directionality, and we cannot exclude the possibility that preclinical or occult tumor processes contributed to the inflammatory profile observed at baseline.

A key contribution of this study is the joint evaluation of metabolic and inflammatory burden. Participants with concomitantly elevated METS-IR and SIRI had the highest risk estimate for RCC (HR: 2.40; 95% CI: 2.06–2.79), suggesting that coexisting metabolic and inflammatory abnormalities may identify a less favorable risk profile [25]. However, additive interaction metrics (RERI, AP, and SI) did not provide statistical evidence of interaction, and the confidence interval for RERI included the null. Therefore, this finding should be interpreted as risk accumulation rather than proven biological or statistical synergy. Nonetheless, the coexistence of metabolic dysregulation and systemic inflammation remains biologically plausible in the context of renal carcinogenesis [26]. Specifically, insulin resistance may amplify inflammatory signaling via NF-κB activation and cytokine release from adipose tissue, while inflammation may in turn exacerbate insulin resistance through mechanisms such as serine phosphorylation of IRS-1, thereby sustaining a pro-carcinogenic milieu [27]. From a public health perspective, these findings support the importance of considering metabolic and inflammatory risk jointly, even though our data do not demonstrate a formal additive interaction [28].

The trajectory analyses are potentially informative but should be considered exploratory. Compared with the Stable Low group, persistently high METS-IR and persistently high SIRI were associated with higher RCC risk, whereas the estimates for the Improved and Worsened groups were imprecise and not all comparisons reached statistical significance. Therefore, these findings should not be interpreted as evidence that normalization of systemic inflammation completely mitigates excess risk, nor that improvement in metabolic status fails to reverse risk because of a confirmed “metabolic memory” effect. Rather, the results suggest that longitudinal patterns of metabolic and inflammatory status may carry different prognostic information. Prior studies have reported that trajectory patterns of metabolic dysfunction may be associated with future cancer risk [29]. In parallel, “metabolic memory” has been proposed as a conceptual framework whereby prolonged metabolic disturbances may leave longer-lasting biological imprints [30], including kidney-related molecular changes [31] and epigenetic alterations observed in other settings of metabolic injury [32]. However, in the context of the present observational analysis, these mechanisms should be regarded only as biologically plausible hypotheses that may help contextualize our findings, rather than conclusions directly established by our data. The observed trajectory patterns may also reflect residual confounding, measurement variability, selection related to repeat assessment attendance, or preclinical disease processes.

The strengths of this study include its substantial sample size, cohort design, and the rigorous exclusion of reverse causality through lag-time analyses. Additionally, the use of repeated biomarker measurements allowed for trajectory modeling, providing temporal insights unavailable in cross-sectional studies [33]. Nevertheless, several limitations should be acknowledged. First, as an observational analysis, residual confounding cannot be excluded despite multivariable adjustment. Information on diet, physical activity, and some other metabolic or inflammatory conditions was not fully incorporated into the final models [34]. Second, differential diagnostic intensity may have influenced the observed associations: participants with higher cardiometabolic burden may undergo more frequent medical evaluation and abdominal imaging, potentially increasing the likelihood of incidental RCC detection independent of a true biological association. Third, the primary analyses relied on a single baseline biomarker assessment, while repeat measures were available only in a subset, making the study vulnerable to measurement error, intra-individual variability, and potential selection bias in the trajectory analyses. Fourth, we were unable to distinguish incidentally detected RCC from symptomatic RCC in the current UK Biobank analysis because mode of presentation was not reliably captured in the linked dataset. Although morphology information was available for most incident kidney cancer cases, histological categories were highly imbalanced, and several records were coded using rare or non-specific morphology labels. Accordingly, histology-specific analyses were restricted to exploratory clear-cell RCC and papillary RCC analyses. Finally, UK Biobank participants are predominantly White and healthier than the general UK population, which may limit external validity [35].

## Conclusion

This UK Biobank cohort analysis showed that METS-IR and SIRI were independently associated with RCC risk, characterized by linear and threshold-dependent dose-response patterns, respectively. We observed a combined high-risk pattern in which simultaneous metabolic and inflammatory dysregulation was associated with higher RCC risk. In exploratory trajectory analyses, persistently high metabolic or inflammatory burden was associated with higher risk, whereas estimates for the improved and worsened groups were imprecise. These findings support early metabolic and inflammatory risk assessment, but the longitudinal results should not be interpreted as evidence of reversibility or direct intervention benefit.

## Supplementary Information

Below is the link to the electronic supplementary material.

Supplementary Material 1

## Data Availability

Data from the UK Biobank (ukbiobank.ac.uk/) are available to all researchers on making an application.
