# Supplementary material for: Joint effects and dynamic trajectories of metabolic insulin resistance and systemic inflammation in the risk of renal cell carcinoma: a UK Biobank cohort analysis
Source: World J Urol. 2026 May 28;44(1):394. doi: 10.1007/s00345-026-06487-x (PMC13219073; doi:10.1007/s00345-026-06487-x)
Supplement: Supplementary file 1 — Supplementary Material 1 [file 345_2026_6487_MOESM1_ESM.docx]

**Figure S1.** Distributional characteristics of metabolic and inflammatory biomarkers stratified by incident renal cell carcinoma status.


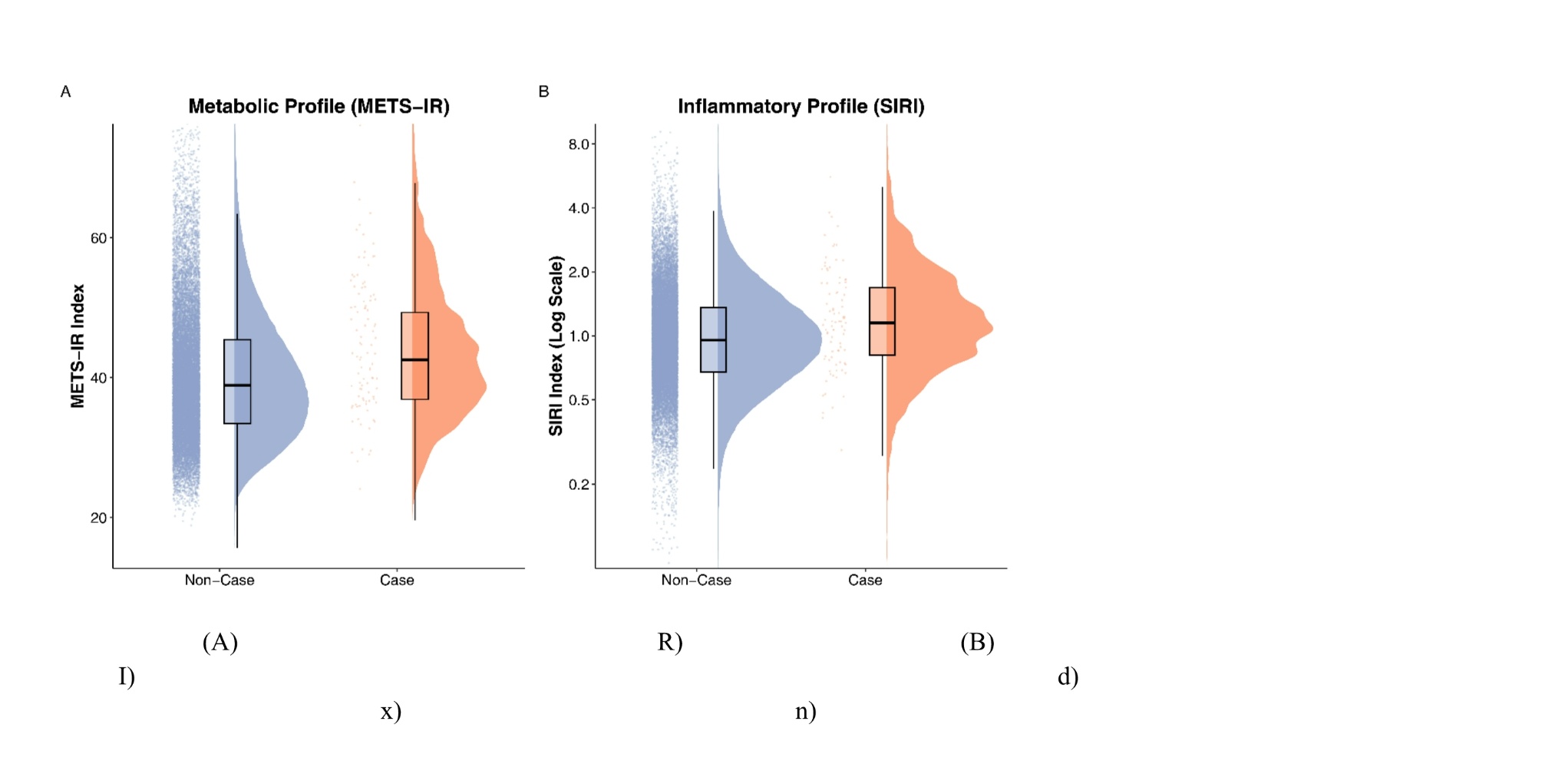


**Figure S2.** Kaplan–Meier curves for cumulative incidence of renal cell carcinoma according to quartiles of METS-IR and SIRI. (A) Comparison of the Metabolic Score for Insulin Resistance, (METS-IR) between non-cases and incident RCC cases., (B) Comparison of the Systemic Inflammation Response Index, (SIRI) between groups, visualized on a logarithmic scale. Each raincloud plot consists of a half-violin plot displaying the probability density, (cloud), a boxplot showing the median and interquartile range, (box), and jittered points representing raw data observations, (rain). The orange color represents the RCC case group, while the blue color represents the non-case group.


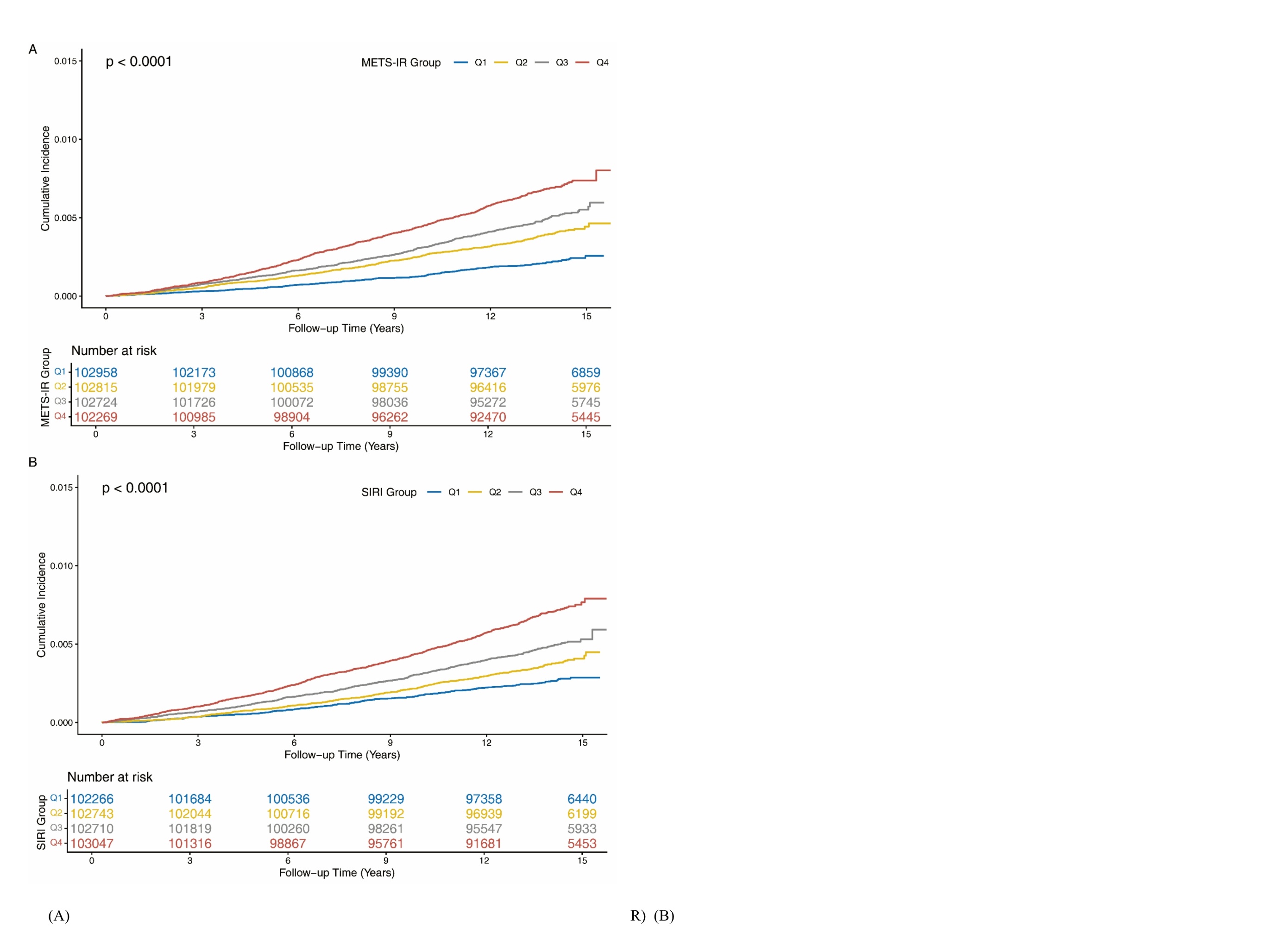


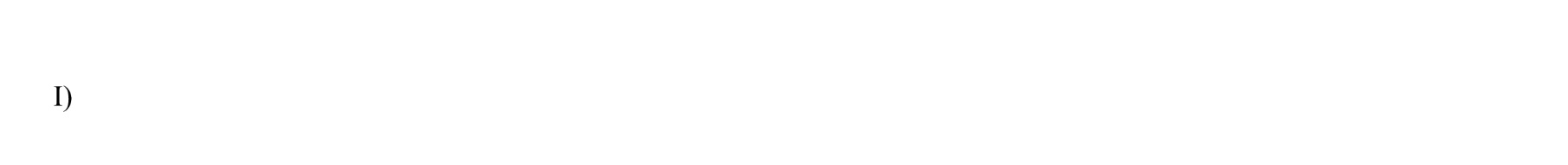


**Figure S3.** Distribution of morphology categories among incident kidney cancer cases with available morphology information. (A) Kaplan–Meier curves showing cumulative incidence of renal cell carcinoma across quartiles of the Metabolic Score for Insulin Resistance, (METS-IR)., (B) Kaplan–Meier curves showing cumulative incidence of renal cell carcinoma across quartiles of the Systemic Inflammation Response Index, (SIRI). Differences between groups were assessed using the log-rank test. For both biomarkers, cumulative incidence increased progressively from Q1 to Q4 over follow-up, (both log-rank P < 0.0001).


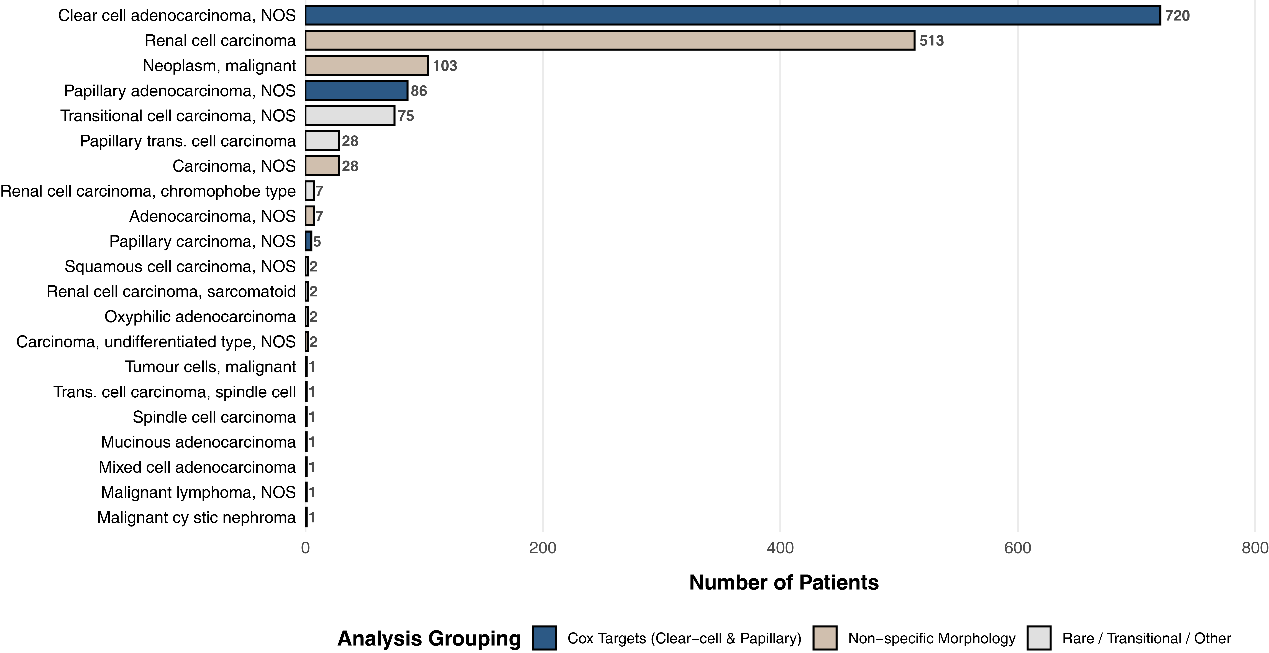


**Table S1. Joint effect and additive interaction analysis of metabolic and inflammatory dysregulation on incident renal cell carcinoma risk.**

| **Classification** | **HR (95% CI)** | **P value** |
| --- | --- | --- |
| **Joint Effect Analysis** |  |  |
| Low-Risk (Reference) | 1.00 (Ref) |  |
| *METS-IR < Median & SIRI < Median* |  |  |
| Metabolic-Risk Only | 1.72 (1.46–2.02) | < 0.001 |
| *METS-IR ≥ Median & SIRI < Median* |  |  |
| Inflammatory-Risk Only | 1.60 (1.36–1.89) | < 0.001 |
| *METS-IR < Median & SIRI ≥ Median* |  |  |
| Double-Hit Group | **2.40 (2.06–2.79)** | **< 0.001** |
| *METS-IR ≥ Median & SIRI ≥ Median* |  |  |
| **Measures of Additive Interaction** | **Estimate (95% CI)** | **Indication** |
| RERI | 0.08 (-0.23 to 0.40) | No significant synergy |
| AP | 0.03 | — |
| SI | 1.06 | — |

**Abbreviations:** HR, hazard ratio; CI, confidence interval; METS-IR, metabolic score for insulin resistance; SIRI, systemic inflammation response index; RERI, relative excess risk due to interaction; AP, attributable proportion due to interaction; SI, synergy index. Analyses were adjusted for age, sex, Townsend deprivation index, ethnicity, smoking status, alcohol consumption, history of hypertension, history of diabetes, use of aspirin and NSAIDs, and baseline eGFR.

**Table S2. Exploratory histology-specific associations of METS-IR and SIRI with incident clear-cell RCC and papillary RCC**

| **Outcome subtype** | **Events, No.** | **Exposure** | **HR** | **95% CI** | **P value** |
| --- | --- | --- | --- | --- | --- |
| Clear-cell RCC | 720 | METS-IR (per 1-SD increase) | 1.44 | 1.35–1.54 | < 0.001 |
|  |  | SIRI (per 1-SD increase) | 1.02 | 1.00–1.04 | 0.300 |
| Papillary RCC | 91 | METS-IR (per 1-SD increase) | 1.04 | 0.84–1.30 | 0.700 |
|  |  | SIRI (per 1-SD increase) | 1.02 | 1.00–1.05 | 0.300 |

*Abbreviations:* CI, confidence interval; HR, hazard ratio; METS-IR, Metabolic Score for Insulin Resistance; RCC, renal cell carcinoma; SIRI, Systemic Inflammation Response Index.

**Table S3. Sensitivity analysis of the associations between metabolic and inflammatory indices and incident renal cell carcinoma risk (excluding baseline malignancy and cases diagnosed within 2 years).**

| **Exposure** | **HR (95% CI)** | **P value** |
| --- | --- | --- |
| **METS-IR** |  |  |
| Per 1-SD increase | 1.29 (1.14–1.46) | < 0.001 |
| *Quartiles* |  |  |
| Q1 (Reference) | 1.00 (Ref) |  |
| Q2 | 1.24 (1.03–1.49) | 0.023 |
| Q3 | 1.23 (1.01–1.50) | 0.039 |
| Q4 | 1.39 (1.09–1.78) | 0.008 |
| *P for trend* |  | 0.024 |
| **SIRI** |  |  |
| Per 1-SD increase | 1.02 (1.01–1.03) | 0.065 |
| *Quartiles* |  |  |
| Q1 (Reference) | 1.00 (Ref) |  |
| Q2 | 1.22 (1.03–1.44) | 0.022 |
| Q3 | 1.31 (1.12–1.54) | 0.001 |
| Q4 | 1.53 (1.31–1.79) | < 0.001 |
| *P for trend* |  | < 0.001 |

**Abbreviations:** HR, hazard ratio; CI, confidence interval; METS-IR, metabolic score for insulin resistance; SIRI, systemic inflammation response index; SD, standard deviation. Sensitivity analysis excluded participants with a history of malignancy at baseline (except non-melanoma skin cancer) and those diagnosed with renal cell carcinoma within the first 24 months of follow-up to minimize reverse causality. Hazard ratios were estimated using the fully adjusted Model 3, controlling for age, sex, Townsend deprivation index, ethnicity, smoking status, alcohol consumption, history of hypertension, history of diabetes, use of aspirin and NSAIDs, and baseline eGFR. Consistent with the main analysis, BMI was included for the SIRI analysis but excluded for the METS-IR analysis.

**Table S4. Sensitivity analysis of the associations of METS-IR and SIRI with incident renal cell carcinoma after additional adjustment for dietary factors and physical activity**

| **Exposure** | **HR (95% CI)** | **P value** |
| --- | --- | --- |
| **METS-IR** |  |  |
| Per 1-SD increase | 1.43 (1.25–1.62) | < 0.001 |
| *Quartiles* |  |  |
| Q1 (Reference) | 1.00 (Ref) |  |
| Q2 | 1.29 (1.06–1.58) | 0.011 |
| Q3 | 1.32 (1.06–1.63) | 0.013 |
| Q4 | 1.63 (1.25–2.13) | < 0.001 |
| *P for trend* |  | 0.024 |
| **SIRI** |  |  |
| Per 1-SD increase | 1.03 (1.02–1.05) | 0.006 |
| *Quartiles* |  |  |
| Q1 (Reference) | 1.00 (Ref) |  |
| Q2 | 1.13 (0.94–1.36) | 0.200 |
| Q3 | 1.34 (1.12–1.60) | 0.001 |
| Q4 | 1.64 (1.38–1.95) | < 0.001 |
| *P for trend* |  | < 0.001 |

**Abbreviations:** CI, confidence interval; HR, hazard ratio; METS-IR, metabolic score for insulin resistance; SIRI, systemic inflammation response index; IPAQ, International Physical Activity Questionnaire; RCC, renal cell carcinoma
